# Representation of drug addiction in Pakistani print media: A Latent Dirichlet Allocation analysis

**DOI:** 10.1371/journal.pone.0356760

**Published:** 2026-09-15

**Authors:** Saad Zahid, Muhammad Nawaz, Kanwal Zahra

**Affiliations:** 1 Department of Humanities, COMSATS University Islamabad, Islamabad Campus, Islamabad, Pakistan; 2 Centre for Languages and Translation Studies, University of Gujrat, Gujrat, Pakistan; Islamic World Science & Technology Monitoring and Citation Institute (ISC), IRAN, ISLAMIC REPUBLIC OF

## Abstract

This paper investigates the representation of drug addiction in Pakistani English print media articles using a topic modelling algorithm known as Latent Dirichlet Allocation. The data, based on 4,100 articles (2014–2023), were collected from the widely circulated newspaper, The News International. The data were retrieved from the LexisNexis database. This research employs Grounded Theory, a data-driven approach that generates explanations or theories from the data. The results showed that drug addiction is presented as interconnected themes, placed in a broader social and institutional context. Drug addiction, particularly among youth, is on the rise and is described as a serious concern. This multifaceted issue is situated at the intersection of public health, social welfare, criminal activity, and national security. Media representation of the issue highlights law enforcement and emphasizes the importance of public awareness, social responsibility, prevention, and rehabilitation of victims, and policy interventions. The findings reveal that state-centric and enforcement-related narratives dominate the themes of treatment, education, and prevention. This study, therefore, recommends a more balanced representation that focuses equally on prevention, treatment, and recovery.

## 1. Background

Drugs are psychotropic compounds that induce the sensation of pleasure or well-being. The World Health Organisation (WHO) defines a drug as any substance that changes the functions of a living organism upon administration [1]. The Control of Narcotic Substances Act (CNSA) defines addiction as: “addict means a person physically or mentally dependent on any narcotic drug or a psychotropic substance or a person who habitually uses narcotic drugs or psychotropic substances” [2]. Pakistan has been exposed to illicit narcotics cultivation, trafficking, and abuse. Pakistan lies in close proximity to Afghanistan, and the region is significantly related to the production and transit of drugs [3]. Drug-related complications lead to 700 deaths daily in Pakistan, accounting for 250,000 deaths annually [4]. The United Nations Office on Drugs and Crime (UNODC, 2013) holds that 6.4 million individuals are using drugs nationwide, with about 4 million cannabis users, followed by heroin and methamphetamine [5]. Anti-Narcotics Force (ANF) claims an aggravation in the issue, with 7 million drug users [6]. Pakistan’s Ministry of Narcotics Control (MNC), with reference to UNODC, reports the number reaching approximately 9 million [7]. This rising trend of drug addiction in the country is an adversity that inspires this study.

The relevant research cited here, to identify the gaps to be filled, is categorized into three types: a) Research on the issue of drug addiction in Pakistan, b) Research involving the application of natural language processing tools on large volumes of textual data, and c) Research on the media representation of the issue of drug addiction. Research about the issue of drug addiction in Pakistan has brought the issue to the forefront from a variety of perspectives, encompassing the motives and impacts of drugs, variation in drug use, and rehabilitation of victims.

Currently, the use of drugs among the skilled and educated class is increasing greatly [8]. In Pakistan, where youth constitute about 64% of the total population, the prevalence of drugs, particularly in academic settings, is a serious concern [9]. Khyber Pakhtunkhwa (KP) province has the greatest prevalence of drug use, with 11% of the population using drugs. Sindh province ranks second with 6.5% drug users. Baluchistan and Punjab provinces have 05 and 04.8% of the population using drugs, respectively [10]. The data stated above indicates that Pakistan is in a grave situation concerning drug addiction, which is a significant threat to its population, particularly the youth. Research about the leading factors behind drug addiction reveals that relational, marital, and parental factors are key causes of participants’ resorting to drug addiction [11]. Besides social and socio-economic crises, and a sense of disappointment, peers’ circle and inquisitiveness are reported to cause drug addiction [12].

Male students are found to be more prone to drugs than females, though the number of female students is increasing significantly [13]. Low literacy level has been associated with drug addiction [14], which in turn is related to criminal behaviour [15]. The unregulated sale of controlled drugs, the incompetence of pharmacists, and inconsistencies in the drug treatment policies [16] have contributed to the aggravation of the problem. Addiction is related to detrimental effects on health as well. Injectable drug users constitute about 38.4% of the total number of people infected with HIV in Pakistan [17].

Factors like peer pressure, family stability, and emotional attachment are found to have a positive impact on the drug treatment process. Self-motivation is also a significant factor [18]. In the context of Pakistan, hurdles in the prevention of drug disorders include psychological and personality challenges, societal hurdles, economic difficulties, and system-level drawbacks [19]. Mental health services in Pakistan have been very insufficient, keeping in view the magnitude of the problem, and the poor regulation of healthcare facilities adds to the misery [20,21]. Moreover, non-compliance with international standards, such as the lack of services from psychiatrists and sociological officers [22], and clinical pharmacists [23] has also been observed.

Concerning the relationship between media and drug addiction, a close connection has been observed, where the media often acts as both a catalyst and a mirror of drug use. Research shows that exposure to drug-related content, particularly on social media [24], and glamorization of drug use can be potential causes of drug addiction. Drug advertisements in the media, both active and passive, are reported to have a role in desensitization of individuals and adoption of drugs [25]. Stigmatization of drug addiction is also found in the media [26], supporting the destructive discourse on drug users [27], although a decreasing trend is reported [28]. Media, on the other hand, also has a positive impact on raising awareness among people regarding drug abuse and addiction. Research conducted in Lahore, Pakistan, reports that due to its widespread use and accessibility, social media is an effective means for spreading awareness and education regarding the hazards of drug use [29].

Regarding the media coverage of drug addiction, existing research shows that the main focus is on drug types, crime and drugs, legislative and medical aspects, and treatment coverage. The language use is reported to be negative, and there is limited representation of harm reduction strategies, necessitating more balanced reporting [30]. A quantitative retrospective content analysis of newspaper articles shows that a significant number of articles had stigmatizing content regarding drug use and involved negative stereotypes about drug users [31]. A systematic review of the studies on the representation of substance abuse on social platforms shows that the common themes presented are health and safety, harms, informative content, advertisements, and use behaviours. The study shows that more than 75% and 20.2% of the content involved positive and negative depictions of drug use, respectively [32] (Rutherford et al., 2023). The newspaper articles on the issue involve a dominant depiction of the personal details of the addicts, highlighting punishment, death, or suicide, rather than recovery or awareness- oriented content. Coverage concerning celebrities portrayed substance use in an encouraging way [33]. A research analysis of the newspaper coverage of drug abuse in Nigeria, newspapers portray the issue as a security threat to the country, lacking the depiction of its health or social consequences [34].

The above-cited research indicates that the issue of drug addiction has been investigated from a variety of perspectives, highlighting the significance of the issue. The cited research shows that media coverage of the issue plays a crucial role in raising awareness and shaping public perception. It is also an important factor in policy-making discussions. Concerning the relationship between media and drug addiction, very few studies have been found in Pakistan. Existing studies generally focus on social media and address isolated themes. Hence, the existing research is mainly thematically narrow, context-specific, and geographically limited, with relatively limited data sets. The representation of the issue in print media remains largely unexplored. Therefore, this research aims to address this gap using a corpus of national English newspaper articles. Using the Grounded Theory method may reveal a clearer, more objective understanding of the issue and its media representation. It may make valuable contributions towards addressing the issue and improving awareness campaigns. Moreover, newspaper articles may cover the issue from a wide range of perspectives, depicting different tendencies and dynamics. Hence, this research aims to identify the dominant themes regarding drug addiction in Pakistani English newspaper articles and analyse the framing of the issue, i.e., how certain aspects of the issue are emphasized, uncovering public and policy implications.

The third type of research we consulted is the published data on natural language processing (NLP) and its various applications. In corpus linguistics, different natural language processing algorithms are applied to achieve a variety of desired outcomes. It is applicable in interpreting, categorising, and processing large volumes of data from different data sources. One of them is sentiment mining [35,36]. The NLP algorithms successfully categorise the reviews into the desired categories with above 80% accuracy. NLP has also been integrated in corpus-based media studies to analyse divergent media perspectives [37]. Classifying scattered text is a crucial problem in digital forensics, and NLP is also effective in this domain [38].

Topic modelling, an NLP tool, has been used by researchers across various fields to analyse large texts by extracting themes and topics [39]. This technique can assist in making systematic literature reviews [40] and extracting topics from significant literary texts [41]. Latent Dirichlet Allocation, a topic modelling algorithm, has been used in computational linguistics to investigate and analyse the print media perspectives on certain issues [42], and for the comparison of the perspectives of print media of different countries on an issue [43]. Cho, Kim, and Woo apply topic modelling to online news articles about women’s health in Korea, revealing that narratives about women’s health change over time [44]. Another research investigated women’s daily life and stressors using topic modelling. The LDA-extracted topics are analysed and reveal multiple stressors, including variance in expected role demands, lack of communication, family load, interpersonal gaps, and others [45]. The research quoted above highlights the wide-ranging scope of natural language processing techniques, particularly in computational research, as well as their effectiveness in analyzing large volumes of text. Therefore, using topic modelling in this study may prove effective in getting a clear picture of the ongoing drug crisis in Pakistan.

## 2. Methodology

This corpus-based research employs both qualitative and quantitative approaches. The corpus of 4100 Pakistani English newspaper articles (2014–2023) was developed from *The* *News International*, using the LexisNexis database. The rationale for selecting this newspaper is that it published more articles on the issue than other national English-language newspapers, and that this time frame may offer the most recent data available. News articles, including the headlines and the detailed text, were selected for analysis. The detailed text provides an in-depth picture of the issue, and topic modelling also works better with large volumes of text. The data analysis was carried out in three steps. The first step involves processing the data; the second step consists of applying LDA to the data, a process that falls under computational linguistics. Finally, the generated topics are analysed and interpreted.

### 2.1. Data collection and pre-processing

On the LexisNexis database, the Boolean operators “Drug* AND Addict* OR Traffick* OR Opium OR Abuse OR Narcotics” were applied to retrieve the data. The other filters that were applied included region (Asia, Pakistan), language (English), source name (The News International), and source type (newspapers). The time frame was set from October 2014 to June 2023. The data was then pre-processed to make it suitable for NLP algorithms. Data pre-processing structures the text data to remove irregularities and redundancies, thereby enhancing the effectiveness and efficiency of topic modelling analyses [46]. Firstly, the noise, such as the headers, footers, HTML, numerical data, and metadata [47] were removed from the corpus to obtain more relevant, purified data using regular expressions. Afterwards, the large text was broken down into distinct, meaningful units [48], called tokens, such as separate words for efficient analysis and interpretation. Thirdly, normalisation of text was carried out. Capitalization, punctuation, and stop words were removed in the first stage of normalization [49]. Standard stop words were removed, and no domain-specific list was constructed. Secondly, the inflections from words were removed to convert the words into their root form [50]. Tokenization and normalization of text were performed in Python using the NLTK (Natural Language Toolkit) library.

### 2.2. Application of Latent Dirichlet Allocation

Latent Dirichlet Allocation (LDA), developed by Blei and others in 2003, is a robust method for analyzing hidden meanings in large-scale unsupervised data [51]. This algorithm regards documents as generated from hidden topics, which are seen as probability distributions over words. It generates keywords and organises them by weight, indicating a keyword’s influence within a group. It enables fast and efficient processing of extensive collections of texts [52]. LDA was applied to generate topics for the current study. For this study, 20 topics were extracted using LDA, each topic consisted of twenty keywords, and clear topic variation across the data was obtained. This was done to avoid the few overly generalized, higher, redundant, and fragmented topics. Then the generated keywords were semantically examined to obtain a central theme from them. Moreover, contextual interpretation of the salient keywords from representative data was carried out. Each keyword cluster was assigned a suitable label inductively, based on the semantic and contextual understanding to ensure accuracy while depicting the hidden thematic patterns in the data. This subjective, interpretive approach was adopted considering the heterogeneous and context-dependent nature of newspaper discourse, where thematic meaning is better captured through semantic interpretation.

### 2.3. Theoretical framework

Grounded Theory theoretically guides this research. It is an inductive, data-driven approach to understanding particular phenomena without previous hypotheses or preconceptions [53]. Using grounded theory, the researcher moves back and forth between the data and analysis, and the conclusions or theory emerge from the data [54]. Nelson terms the application of computational tools like LDA in grounded theory research as Computational Grounded Theory, which can be called an upgraded version of Grounded Theory [55]. This modern approach to grounded theory combines human knowledge and hermeneutic expertise with the data-processing and pattern-recognition power of computers. This process includes computer-based pattern detection, pattern refinement aided by human knowledge, and pattern confirmation that ultimately uncovers the meaning grounded in the data. In line with grounded theory principles, the LDA-extracted keyword clusters were labelled with suitable themes/topics, and these topics were refined through iterative comparison across the data. The related topics are organized into broader categories, and finally, dominant narrative structures in the newspaper’s coverage of drug addiction were identified. Thematic saturation was achieved by evaluating thematic stability across model iterations. Hence, a computer-assisted approach to Grounded Theory is employed in this research.

## 3. Analysis and interpretation of the data

Table 1 shows the topics extracted from the data using LDA. The keywords generated through LDA are presented in the left column. The keyword groups have the words arranged in descending order of weightage. Higher weightage indicates a more decisive influence and significance of a keyword in that group. The right column shows the themes assigned to the keywords.

**Table 1:** LDA generated keywords and suggested themes.

No.	Keywords	Themes
0	‘0.033*“drug” + 0.029*”student” + 0.020*”youth” + 0.018*”society” + “0.014*”address” + 0.011*”education” + 0.010*”university” + ‘ ‘0.010*”government” + 0.010*”addiction” + 0.009*”occasion” + 0.009*”issue” + “0.009*”event” + 0.008*”people” + 0.008*”need” + 0.008*”training” + “0.008*”school” + 0.008*”support” + 0.008*”highlight” + 0.008*”menace” + “0.008*”programme”	Addiction in youth
1	‘0.296*“drug” + 0.080*”use” + 0.078*”addict” + 0.044*”narcotic” + “0.022*”anf” + 0.020*”anti_narcotic” + 0.018*”supply” + 0.017*”coordination” “+ 0.017*”cocaine” + 0.015*”opium” + 0.013*”afghan_national” + “0.012*”produce” + 0.011*”harm” + 0.011*”capacity_builde” + 0.011*”sell” + “0.011*”menace” + 0.009*”federal_capital” + 0.007*”curb” + “0.007*”assistance” + 0.007*”lab”	Reforms for narcotics control
2	‘0.223*“police” + 0.065*”arrest” + 0.046*”accuse” + 0.034*”recover” + “0.033*”case” + 0.023*”police_station” + 0.017*”register” + “0.017*”possession” + 0.017*”cop” + 0.016*”involve” + 0.016*”investigation” “+ 0.012*”city” + 0.012*”crime” + 0.011*”arrested_accuse” + 0.011*”weapon” + “0.011*”team” + 0.010*”robbery” + 0.010*”district” + 0.009*”lahore_police” + “0.009*”include”	Law enforcement activities of police
3	‘0.037*“zafar” + 0.031*”akram” + 0.031*”highway” + 0.030*”long_time” + “0.028*”ejaz” + 0.013*”absconder” + 0.013*”survey_conducte” + “0.011*”manhole” + 0.010*”municipal” + 0.009*”loudspeaker” + 0.007*”char” + “0.007*”ameen” + 0.007*”farooq” + 0.007*”large_amount” + 0.006*”metropolis” “+ 0.005*”seventh” + 0.005*”mohamme” + 0.003*”stolen_valuable” + “0.002*”traveller” + 0.002*”satellite”	Drugs linked with thefts and robberies
4	‘0.033*“government” + 0.018*”power” + 0.015*”country” + 0.012*”leader” + “0.012*”political” + 0.012*”war” + 0.012*”attack” + 0.012*”business” + “0.011*”money” + 0.011*”demand” + 0.011*”terrorist” + 0.011*”terrorism” + “0.009*”illegal” + 0.009*”rule” + 0.009*”corruption” + 0.008*”crime” + “0.008*”example” + 0.008*”worker” + 0.008*”state” + 0.007*”million”	Terror financing and drug trade
5	‘0.015*“make” + 0.012*”people” + 0.012*”time” + 0.011*”even” + “0.010*”become” + 0.008*”well” + 0.007*”take” + 0.007*”go” + 0.007*”give” + “0.006*”life” + 0.006*”way” + 0.006*”work” + 0.006*”good” + 0.006*”lead” + “0.005*”change” + 0.005*”new” + 0.005*”get” + 0.005*”know” + 0.005*”use” + “0.005*”first”	Emphasis on societal efforts to address addiction
6	‘0.101*“tobacco” + 0.072*”smoke” + 0.040*”ruin” + 0.031*”smoking” + “0.026*”prohibition” + 0.016*”restaurant” + 0.014*”ban” + “0.010*”tobacco_smoke” + 0.008*”shop” + 0.008*”harmful” + 0.005*”cafe” + “0.000*”tobacco_kill” + 0.000*”cigarette” + 0.000*”nicotine” + “0.000*”smoker” + 0.000*”quit” + 0.000*”gum” + 0.000*”tobacco_product” + “0.000*”lozenge” + 0.000*”medicine”	Measures to minimize smoking
7	‘0.118*“sho” + 0.035*”protest” + 0.034*”massive” + 0.032*”relative” + “0.022*”irrespective” + 0.022*”protester” + 0.020*”upper” + 0.020*”latter” + “0.015*”floor” + 0.014*”explosion”0 + 0.012*”punjab_assembly” + 0.011*”ram” + “0.011*”demonstration” + 0.010*”wind” + 0.007*”traffic_jam” + “0.004*”garment” + 0.004*”storm” + 0.003*”grapple” + 0.003*”two_hour” + “0.003*”water_tank”	Public voice against drugs
8	0.052*“drug_addict” + 0.036*”facility” + 0.034*”prisoner” + 0.031*”centre” “ + 0.030*”project” + 0.028*”rehabilitation” + 0.025*”chief_minister” + “0.025*”provide” + 0.024*”hospital” + 0.022*”treatment” + 0.022*”prison” + “0.021*”province” + 0.018*”prepare” + 0.016*”jail” + 0.016*”construction” + “0.013*”provincial” + 0.013*”establish” + 0.012*”peshawar” + 0.011*”land” + “0.011*”nab”	Infrastructure development
9	‘0.062*“locality” + 0.049*”deprive” + 0.028*”wall” + 0.019*”gas” + “0.018*”quarrel” + 0.017*”opium_kg” + 0.016*”sarfraz” + 0.013*”irfan” + “0.012*”office_bearer” + 0.010*”bandit” + 0.008*”district_w” + “0.005*”thrash” + 0.005*”vain” + 0.004*”extort” + 0.003*”gunshot” + “0.003*”swindler” + 0.003*”wapda” + 0.002*”accomplice” + 0.001*”wooden” + “0.001*”sdo”	Addiction and Crimes
10	‘0.058*“educate” + 0.049*”injection” + 0.041*”background” + “0.035*”various_area” + 0.029*”raids_conducte” + 0.029*”canal” + “0.023*”litres_liquor” + 0.016*”old_enmity” + 0.013*”rashee” + 0.011*”slum” “ + 0.009*”iftikhar” + 0.008*”people_live” + 0.006*”foiled_bid” + “0.006*”postmortem” + 0.005*”brb_canal” + 0.004*”unidentifie” + “0.003*”smuggle_huge” + 0.003*”villager” + 0.002*”amin” + ‘	Community education to prevent addiction
11	‘0.023*“security” + 0.016*”country” + 0.015*”region” + 0.013*”international” “+ 0.012*”cooperation” + 0.011*”project” + 0.011*”border” + 0.010*”include” “0.010*”afghan” + 0.010*”support” + 0.010*”peace” + 0.010*”effort” + “0.010*”policy” + 0.009*”state” + 0.009*”pakistan” + 0.009*”delegation” + “0.009*”economic” + 0.009*”trade” + 0.009*”discuss” + 0.008*”challenge”	Border management with Afghanistan
12	‘0.020*“official” + 0.018*”officer” + 0.017*”direct” + 0.013*”order” + “0.013*”meeting” + 0.013*”office” + 0.012*”take” + 0.012*”action” + “0.012*”punjab” + 0.011*”city” + 0.011*”district” + 0.010*”regard” + “0.010*”citizen” + 0.010*”issue” + 0.010*”make” + 0.010*”sindh” + “0.009*”add” + 0.009*”ensure” + 0.009*”law” + 0.008*”narcotic”’),	Executive communications about the enforcement of laws for drug control
13	‘0.059*“case” + 0.029*”court” + 0.017*”charge” + 0.015*”evidence” + “0.015*”accuse” + 0.011*”claim” + 0.011*”allege” + 0.010*”witness” + “0.010*”record” + 0.010*”statement” + 0.010*”state” + 0.010*”report” + “0.009*”judge” + 0.009*”law” + 0.009*”drug” + 0.009*”file” + 0.009*”justice” “+ 0.008*”application” + 0.008*”release” + 0.008*”seek”’),	Judicial proceedings
14	‘0.021*“increase” + 0.017*”use” + 0.016*”people” + 0.016*”country” + “0.015*”include” + 0.014*”report” + 0.012*”percent” + 0.012*”year” + “0.011*”number” + 0.011*”health” + 0.010*”need” + 0.010*”due” + “0.009*”treatment” + 0.009*”available” + 0.008*”provide” + 0.007*”disease” + “0.007*”add” + 0.007*”accord” + 0.007*”per_cent” + 0.006*”high”’),	Increasing trend of narcotics use
15	‘0.055*“body” + 0.034*”identify” + 0.028*”kill” + 0.028*”shift” + “0.027*”police” + 0.025*”injure” + 0.025*”die” + 0.022*”victim” + “0.021*”area” + 0.016*”hospital” + 0.016*”murder” + 0.015*”man” + “0.015*”fire” + 0.014*”suspect” + 0.014*”drug” + 0.012*”spot” + “0.012*”addict” + 0.012*”incident” + 0.009*”limit” + 0.009*”death”’),	Deaths linked with drug use
16	‘0.037*“arrest” + 0.031*”drug” + 0.027*”narcotic” + 0.026*”suspect” + “0.022*”seize” + 0.022*”operation” + 0.018*”area” + 0.017*”heroin” + “0.017*”vehicle” + 0.014*”add” + 0.014*”recovery” + 0.014*”peshawar” + “0.013*”involve” + 0.013*”official” + 0.012*”raid” + 0.011*”recover” + “0.011*”carry” + 0.011*”hashish” + 0.011*”involved” + 0.010*”accord”	Operations for drugs control
17	0.054*“child” + 0.048*”woman” + 0.027*”family” + 0.021*”girl” + “0.017*”take” + 0.015*”parent” + 0.014*”victim” + 0.012*”add” + 0.012*”day” “ + 0.011*”fir” + 0.011*”report” + 0.011*”tell” + 0.010*”find” + 0.010*”home” “ + 0.009*”live” + 0.009*”drug_addict” + 0.009*”go” + 0.009*”house” + “0.009*”work” + 0.009*”daughter”’	Domestic and social sufferings
18	‘0.106*“fia” + 0.073*”mortuary” + 0.013*”bogus” + 0.013*”would_soon” + “0.012*”qualification” + 0.008*”substandard” + 0.007*”mp” + “0.005*”motorway_police” + 0.002*”fia_immigration” + 0.000*”cell_ahtc” + “0.000*”inquiry” + 0.000*”medicine” + 0.000*”cognisable” + 0.000*”drap” + “0.000*”referral” + 0.000*”karachis” + 0.000*”camp_jail” + “0.000*”money_laundere” + 0.000*”found_dead_near” + 0.000*”phase”	Law enforcement activities related to drug regulation
19	‘0.030*“parcel” + 0.029*”landikotal” 0.027*”grams_heroin” + “0.019*”drug_supplier” + 0.017*”swabi” + 0.017*”boarding” + “0.017*”secret_cavitie” + 0.016*”sheikh” + 0.009*”cns_act” + 0.008*”baggage” “+ 0.002*”grams_chara” + 0.002*”namely_muhammad” + 0.000*”hangu” + “0.000*”ice” + 0.000*”excise” + 0.000*”sniffer” + 0.000*”election” + “0.000*”package” + 0.000*”appellant” + 0.000*”smuggle_drug”	Narcotics trafficking

The first set of keywords in the table, that is, topic 0, revolves around the issue of drug addiction in youth, particularly in academic institutions. The keywords, such as ‘student’, ‘youth’, ‘education’, ‘university’, and ‘school’, lead to this inference. There is a need to eradicate the menace through various programs and initiatives. Topic 1 reflects on institutional reforms to control the spread of narcotics in Pakistan. The themes like ‘capacity building’ of the institutions like Anti Narcotic Force, and ‘coordination’ between various law enforcement institutions to ‘curb’ drug addiction are discussed. Moreover, the construction of modern infrastructure, such as ‘labs’, is also mentioned as part of these reforms in this topic. Topic 2 exhibits the law enforcement activities of police. It signifies the involvement of police teams in taking measures to counter drug-related crimes, like illegal drug possession, intake, or trafficking, involving ‘arrests’ and ‘recovery’ of illicit drugs, or carrying out ‘investigations’. Secondly, drugs linked with other offences like ‘robberies’, and illegal possession and use of ‘weapons’ in such incidents are highlighted.

The topic 3 thematically overlaps with the theme in topic 2, that is, drug addiction linked with other crimes like thefts and robberies. The keywords like ‘absconder’, ‘large amount’, and ‘stolen valuable’ indicate such offences. This also includes the stealth of the ‘manhole’ covers, often sold by addicts to purchase drugs. Topic 4 relates to crime and terrorism, its relationship with drug trafficking in the region, and the involvement of the ‘government’ to counter this. Drug trafficking and terrorism are interlinked and are depicted as national concerns. Drug trafficking is one of the illegal ‘businesses’, spread worldwide to fund terrorist activities. The keywords ‘money’ and ‘million’ suggest a focus on the role of the illicit drug trade that funds and sustains the terror organisations. Topic 5 includes general, rather than specific, but interpretable terms about the issue of drug addiction and abuse in Pakistan. These terms collectively contribute to the required efforts on a societal level to overcome the detrimental effects of drug use. The keywords like ‘make’, ‘work’, and ‘give’ suggest the need to ‘take’ practical measures on a societal level that will ‘lead’ to a positive ‘change’ in society. The keyword ‘people’ suggests the human aspect of the issue, referring to both the affected people and society, to give proper attention, time, and resources to support the drug victims. The keywords ‘become’, ‘well’, and ‘good’ reflect the desired positive outcomes of these efforts, indicating the goal of improving the ‘life’ of the affected individuals and, hence, a good society.

Topic 6 centres on the inadequate enforcement of regulations concerning the purchase and use of tobacco products. It also emphasizes the need to strictly enforce rules regarding tobacco purchase and use. This is indicated by words like ‘tobacco’, ‘prohibition’, ‘ban’, ‘restaurant’, and ‘shop’. Moreover, an emphasis is laid on the harmful effects of tobacco administration through keywords like ‘ruin’, ‘harmful’, ‘tobacco kills’, and the ways for giving up smoking by keywords like ‘quit’, ‘gum’, ‘lozenge’, and ‘medicine’ are discussed. The list of words in topic 7 presents public voices against drugs. This is done through ‘protests’ and ‘demonstrations’, urging the government to ‘grapple’ with the flow of illicit drugs in the country. Topic 8 refers to the government’s actions to provide better health facilities for the treatment and rehabilitation of drug victims, including those imprisoned. The keywords like ‘facility’, ‘centre’, ‘project’, ‘hospital’, ‘treatment’, ‘construction’, and ‘establish’ lead to the inference as mentioned above.

Topic 9 depicts the same theme as topic 3, i.e., the incidents like extortion, violence, and other fraudulent practices linked with drugs. The words like ‘quarrel’, ‘thrash’, and ‘gunshot’ suggest the violence taking place, while ‘deprive’, ‘bandit’, ‘extort’, and ‘swindler’ show the occurrence of thefts and robberies. Community education, along with strong law enforcement activities, is regarded as a way to prevent addiction in topic 10. ‘Slums’ are reported to have been associated with the issue of drugs due to a lack of education and development. The Pakistan-Afghanistan border is reported to be creating serious ‘security’ risks and is regarded as a route for drug trafficking. Hence, in topic 11, effective border management with Afghanistan, internally through policymaking, and externally through dialogue, is stressed. The keywords ‘region’, ‘cooperation’, ‘delegation’, and ‘discuss’ suggest the need for mutual coordination and dialogue to address this issue. The border fencing ‘project’ along the Pakistan-Afghanistan border is termed as a positive initiative.

The subject discussed in topic 12 is executive communications and directions about the enforcement of laws for controlling drug addiction and related issues. The keywords like ‘official’, ‘officer’, ‘direct’, ‘order’, ‘meeting’, ‘action’, and ‘ensure’ suggest the meaning mentioned before. While topic 2 presents the active role of law enforcement institutions, such as the police, in addressing drug-related offences, topic 13 depicts the next stage, i.e., judicial proceedings. The activities related to the legal processes and trials against those charged with drug-related offences are in focus. The keywords like ‘case’, ‘court’, and ‘charge’ are central to the matter and represent the legal processes involved in bringing them to ‘justice’. The same meaning is depicted by the words ‘witness’, ‘record’, ‘statement’, ‘application’, ‘file’, and ‘release’. An increasing trend of drug addiction is suggested in topic 14. This inference is supported by the keywords like ‘increase’, ‘use’, and ‘high’. The words like ‘report’, ‘number, and ‘percent’ refer to the statistics and figures issued by relevant organisations and institutions, or monthly/yearly reports and figures issued by other law enforcement agencies.

Topic 15 exhibits the deaths linked with the use of drugs, resulting from both drug overdose and violence. The words ‘body’, identify’, ‘shift’, drug’, and ‘addict’ refer to the deaths linked with drugs, and the words ‘kill’, ‘injure’, and ‘murder’ indicate the violent incidents. Topic 16 overlaps with Topic 2, as it also shows the activities of law enforcement agencies. The word ‘operation’ conveys the central idea. Other words, like ‘arrest’, ‘drug’, ‘narcotic’, ‘seize’, ‘raid’, and ‘recover’, denote the activities under these operations. Domestic and social sufferings due to drug addiction and abuse are discussed in topic 17.

The most prominent words ‘child’, ‘woman’, ‘family’, ‘parent’, ‘drug addict’, and ‘daughter’ refer to the most likely ‘victims’ of the effects of drug addiction. The words like ‘tell’, ‘fir’, and ‘report’ reflect the interventions of law enforcement institutions after such offences. Topic 18 outlines the activities of law enforcement agencies, such as the Federal Investigation Agency (FIA) and the Drug Regulatory Authority of Pakistan (DRAP), related to drug regulation. In this context, the keyword ‘qualification’ suggests the failure to meet the required standards. This has strong connections with the manufacturing of the ‘substandard’ medicines. Topic 19 sheds light on drug trafficking. Drugs are trafficked in the guise of ‘parcels’, in ‘baggage’, as well as in ‘secret cavities. The words like ‘grams heroin’, ‘ice’, and ‘grams charas’ describe various drugs, while ‘landikotal’, ‘swabi’, and ‘hangu’ denote the geographical context of such activities.

## 4. Discussion

This research investigated the themes reflected in Pakistani print media on the issue of drug addiction. The themes generated by LDA from newspaper data are grounded in the text. This approach allowed for the identification of latent themes in newspaper coverage and their interpretation in relation to broader social and institutional meanings. These themes highlight that drug addiction is depicted as a systemic, multifaceted issue, and the need for timely and effective measures to avoid this issue is emphasized, indicating the existence of loopholes on multiple sides. The results reveal that the Pakistani media intends to warn its readers and authorities about the severity of the problem.

The LDA-generated topics indicate that media discourse revolves around many interconnected dimensions. These include increased prevalence of drug use, particularly among youth, the social [56], familial [57,58] and national consequences of substance abuse, drug trafficking, and institutional responses by law enforcement agencies. High prevalence of drugs among youth, particularly in academic settings [59], appears as a dominant pattern in media representation. This reflects concern over the vulnerability of youth and thus positions drug addiction as a threat to the country’s human capital, rather than just a health issue. Drug trafficking, resulting in increased availability, is represented as one of the significant reasons behind the widespread of drug addiction in the country. Terror financing through drug trade [60] is also a recurrent theme. Thus, domestic consumption is linked to both internal and cross-border criminal networks. As a result, national security and public health are compromised [61]. Hence, this association places the issue within a broader security discourse. The impacts of drug addiction that span across a variety of other dimensions are discussed. Domestic conflicts, violence, and abuse due to drugs [62] are particularly highlighted in Pakistani news articles. A focus on law enforcement activities suggests that the problem is worsening in Pakistan. Having the widespread use of drugs and consequently, an increased rate of drug-related offences [63] in the context of the state, institutions like the Anti-Narcotics Force, FIA, and police are represented as actively involved in efforts to control the illicit drug production, use, trade, and trafficking. Executive communications involving directives and strategies, the dissemination of figures, and regulatory actions reflect the active involvement of law enforcement institutions. These efforts, however, are deemed insufficient to completely eradicate the issue. The law enforcement institutions are faced with a number of challenges, such as a lack of resources and insufficient training, among others [64]. Hence, the media narratives implicitly call for more effective governance strategies, including capacity building of personnel, enhanced capabilities [65] and coordination among institutes. Effective border management has always been crucial to counter the drug addiction by preventing drug trafficking, and hence, appears as a recurrent theme. This positions drug addiction as a transnational problem, requiring domestic enforcement as well as regional cooperation and strengthened border management [66,67]. In addition to the state-centric narrative, societal efforts in the form of protests and demonstrations are highlighted on one side, and societal role [68] and responsibility in the prevention of drugs and rehabilitation of drug victims on the other side, are regarded as of great significance. The lack of education regarding drug addiction is evident from the media discourses. On the other hand, the discourses also depict the adoption of harm reduction strategies such as nicotine replacement therapy [69] to avoid smoking. These themes, however, remain marginal in comparison with the state centric and enforcement oriented narratives [70,71] within Pakistani print media. Hence, this study suggests strengthening drug awareness and prevention programs, alongside structural improvements and enforcement approaches. Media coverage could focus more on prevention, treatment and recovery [72].

While this study offers useful insights into the representation of the issue of drug addiction in Pakistani newspaper articles, we also acknowledge certain limitations. This analysis is based on the interpretation of data from a single source, which causes restrictions in the ability to compare the phenomenon across different media outlets. Relying on single data source may also introduce bias in representing the issue. Additionally, study is subject to algorithmic constraints in the topic modelling technique. Future research may extend this analysis by incorporating more data sources as well as conducting a trend analysis of topic prevalence over time.

## 5. Conclusion

This research uses LDA to find out the narratives of Pakistani English print media from 2014 to 2023, regarding the issue of drug addiction in Pakistan. The findings indicate that drug addiction is portrayed as a multifaceted issue encompassing public health concerns, social disruption, criminal activity and national security challenges. Drug addiction is placed in broader social and institutional contexts. Particular emphasis is laid on increasing prevalence of drugs among youth, drug trafficking, social and familial consequences of drug addiction, and the activities of law enforcement agencies. Media coverage also highlights the significance of public awareness, preventive and rehabilitation efforts, and policy interventions. Taken together, the findings reveal that the issue is represented through interconnected themes. The study contributes to the understanding of media narratives through which the problem is communicated to the public. Methodologically, this study demonstrates the useful integration of topic modelling with inductive, interpretive approaches to large textual datasets. Future research may extend this work by including multiple media sources and changes in media representation over time to provide an understanding of the evolving discourse surrounding drug addiction.
