# Supplementary material for: Representation of drug addiction in Pakistani print media: A Latent Dirichlet Allocation analysis
Source: PLoS One. 2026 Sep 15;21(9):e0356760. doi: 10.1371/journal.pone.0356760 (PMC13577474; doi:10.1371/journal.pone.0356760)
Supplement: Supplementary file 1 [file pone.0356760.s002.docx]

**Responses to Reviewers**

**Reviewer#1**

| **Comment Number** | **Reviewer’s Comments** | **Responses** |
| --- | --- | --- |
| **1** | Title: The title is not sufficiently precise. The term “discourses” is vague in this context. Since the study analyzes news content from newspapers, more accurate terminology should be used, such as “Pakistani English newspaper articles” or “news coverage”, to clearly reflect the data source and scope. | The changes have been made accordingly. The title has been changed to “Representation of Drug Addiction in Pakistani Print Media: A Latent Dirichlet Allocation Analysis” |
| **2** | Problem statement: The problem statement is not well articulated. The rationale for conducting this study and the specific research gap it addresses are insufficiently explained. | After revision, the problem statement and research gap addressed by this research are articulated clearly: the existing research is mainly thematically narrow, context-specific, and geographically limited, with relatively limited data sets. Moreover, the representation of the issue in print media remains largely unexplored. |
| **3** | Literature review: The review of prior studies is weak and lacks focus. The cited literature should be more closely aligned with the study’s title and objectives, particularly prior research on media representations of drug addiction and computational text analysis or topic modelling in media studies. | The literature review section has been revised. The previous studies on the issue have been critically examined. The studies concerning media representation of drug addiction, and the use of topic modelling and LDA in large text analysis, have been added. |
| **4** | Study objectives: The objectives are stated in an overly general manner. The authors should clearly and explicitly define the study's analytical purpose. | This research aims to identify the dominant themes regarding drug addiction in Pakistani English newspaper articles and analyse how the issue is framed, uncovering public and policy implications. |
| **5** | Methodology: The Methods section is incomplete and lacks transparency. Critical methodological details are missing, which undermines reproducibility and rigor.  - Use of Grounded Theory: The application of Computational Grounded Theory alongside topic modeling is not clearly explained. It is unclear how Grounded Theory principles are operationalized in this study and what analytical role they play beyond LDA. | Grounded theory, in its essence, is the data-driven approach that derives the theory/explanation out of data without any preconceptions. In the current research, the use of LDA, being a data-driven approach, aligns with this main principle of grounded theory. Furthermore, the extracted keywords are organized into broader categories by assigning suitable themes/labels. Like the grounded theory method, these themes are refined by reiterative comparisons across the data. Likewise, thematic saturation is achieved by thematic stability across model iterations. These are some of the core principles of grounded theory operationalized in this research. |
| **6** | Topic modeling and preprocessing details: Although the general steps of preprocessing and LDA are mentioned, several essential methodological aspects are not reported:  -- Which parts of the news articles were analyzed (titles, summaries, full texts, or a combination)? | It has been mentioned that complete articles, including the headlines and the full text, are analysed. |
| **7** | What specific preprocessing steps were performed (e.g., tokenization, lemmatization, stemming, normalization)? | The data pre-processing techniques used are noise removal, normalisation, and tokenization. |
| **8** | How was the number of topics (K = 20) determined? Were quantitative metrics such as coherence scores or perplexity used? | The number of topics was determined through repetitive refinement of the LDA model. Different models were tested, and the final number was selected because it produced clearly differentiated topics shown by meaningful keyword weight distributions across topics. This showed that the selected number of topics were distinct and non-overlapping. On the other hand higher topic number produces fragmented and redundant sub-topics. |
| **9** | -- How were stop words defined? Was a domain-specific stop-word list related to drug addiction constructed? | The standard stop words were removed without constructing a domain-specific word list. |
| **10** | Which software or libraries were used (e.g., Gensim, Mallet, scikit-learn)? | After noise removal using regular expressions, further data pre-processing was carried out in Python using the Natural Language Toolkit (NLTK) library. |
| **11** | How were the topic clusters interpreted and labeled? | Keyword clusters were semantically examined, and keywords were contextually interpreted from representative data, and clusters were labelled based on the interpretation. |
| **12** | Results: The findings are very limited. Given that the dataset spans a 10-year period, the authors should strengthen the Results section by including a temporal or trend analysis of topic prevalence. | We recognize that a temporal or trend analysis would strengthen the results section and expand it, but the objective of this study is to capture the representation of the issue in newspaper articles and to uncover the public and policy implications. Hence, the study examines the prevalence of the topic over a 10-year period. Future research could, however, expand this study by carrying out a trend analysis of topic prevalence over time. |

**Reviewer #2:**

| **Comment Number** | **Reviewer’s Comments** | **Responses** |
| --- | --- | --- |
| **1** | The article is significant and methodologically innovative. However, several points require attention:  Data Source Limitation: Reliance on The News International alone may introduce bias. Including other newspapers or media sources would strengthen the validity of findings. | We agree that incorporating multiple newspapers or media sources would strengthen the validity and comparative robustness of the findings; however, given the time and resource-intensive requirements for corpus preparation, pre-processing modelling, and topic validation, the study is limited to one data source. We acknowledge that this study may introduce bias, and this limitation has been explicitly stated in the revised manuscript. Future studies can extend the analysis by adding more newspapers and other data platforms. |
| **2** | Transparency of Limitations: The manuscript does not clearly state its limitations (e.g., single source, lack of comparison, algorithmic constraints). Explicit acknowledgment would improve credibility. | We acknowledge the need to state these limitations explicitly, and they have been clearly articulated in the revised manuscript. |
| **3** | Parameters and Validation: More detail is needed on the selection of topic numbers for LDA and the validation criteria. | The number of topic models was set to 20 to avoid overly general themes when the number of topics was small and redundant or fragmented themes when the number of topics was large. Based on the semantic interpretation and contextual examination of keywords from representative data, the accuracy of the results was ensured. |
| **4** | Background Section: The background should be more analytical, engaging critically with existing literature and highlighting the research gap. | In the revised version, the background section engages critically with the cited research, highlighting the gap. It has been found that the existing research is mainly thematically narrow, context-specific, and geographically limited, with relatively limited data sets. Moreover, the representation of the issue in print media remains largely unexplored. |
| **5** | Future Research: The study could suggest future directions, such as incorporating social media data or other digital platforms. | Future research directions have been mentioned in the revised manuscript, including a temporal/trend analysis of topic prevalence over time and the inclusion of data from other platforms. |
