# Supplementary material for: Representation of drug addiction in Pakistani print media: A Latent Dirichlet Allocation analysis
Source: PLoS One. 2026 Sep 15;21(9):e0356760. doi: 10.1371/journal.pone.0356760 (PMC13577474; doi:10.1371/journal.pone.0356760)
Supplement: Supplementary file 2 [file pone.0356760.s003.docx]

**Response to Reviewers**

**Reviewer # 3**

| **Comment Number** | **Reviewer’s Comment** | **Response** |
| --- | --- | --- |
| **01** | This research article examines how Pakistani English print media framed the issue of drug addiction based on the data selected between 2014 and 2023.the authors’ use of topic modelling algorithm i.e., Latent Dirichlet Allocation (LDA)was appropriate for the research question that they tried to answer. The findings highlight a growing national concern for regarding substance abuse among youth, its connection to criminal behaviour and terrorism, and the ongoing efforts of law enforcement agencies.  Some issues noted:  If there is one issue I can point out to for improvement, it is the issue of methodological choice of subjective topic selection. That is to say that the number of topics was determined through repetitive refinement and semantic examination rather than using specific quantitative metrics like coherence scores or perplexity- but that is not a major issue. | We acknowledge that LDA-based studies generally use statistical metrics such as perplexity or coherence scores for this purpose. A subjective, interpretive approach was used, given the context-dependent and heterogeneous nature of newspaper discourse, where meaning is better captured through semantic interpretation. |

**Reviewer # 4**

| **Comment Number** | **Reviewer’s Comments** | **Responses** |
| --- | --- | --- |
| **01** | The keywords should be standardised with the Mesh thesaurus. | We appreciate the input. Changes have been made where applicable. |
| **02** | Mention the added value of doing this in the problem statement | The recommended revisions have been made. Considering that the representation of drug addiction in Pakistani print media remains largely unexplored, and the existing body of literature is generally narrow in terms of context, geographically limited, and addresses isolated themes related to addiction. Therefore, this study may contribute to a more comprehensive understanding of the print media representation of the issue and may inform policy considerations. |
| **03** | Considering the findings, the discussion of the work is not very well written and it would be better to have many examples of the studies. | The discussion section has been revised substantially to improve clarity and analytical depth. Additionally, more recent relevant examples have been added to situate the findings within the existing literature, as per the recommendations. |
| **04** | Also correct the conclusion and add suggestions. | The conclusion has been revised, and suggestions have been added. |
